# Development of cool and hot theory of mind and cool and hot inhibitory control abilities from 3.5 to 6.5 years of age

**DOI:** 10.1371/journal.pone.0262251

**Published:** 2022-01-27

**Authors:** Manali Draperi, Ania Aïte, Mathieu Cassotti, Lorna Le Stanc, Olivier Houdé, Grégoire Borst

**Affiliations:** 1 LaPsyDÉ, Université de Paris, CNRS, Paris, France; 2 Institut Universitaire de France, Paris, France; Public Library of Science, UNITED KINGDOM

## Abstract

Attributing affectively neutral mental states such as thoughts (i.e., cool theory of mind, cool ToM) to others appears to be rooted in different processes than the ones involved in attributing affectively charged mental states such as emotions (i.e., hot ToM) to others. However, no study has investigated the developmental pattern of hot and cool ToM abilities using a similar task and the relative contribution of cool and hot inhibitory control (IC) to cool and hot ToM development. To do so, we tested 112 children aged 3.5 to 6.5 years on a cool and a hot version of a ToM task and on a cool and hot version of an IC task. We found that hot ToM abilities developed more rapidly than cool ToM. Importantly, we found that hot IC abilities mediated the relation between age and hot ToM abilities. Taken together, our results suggest that the ability to attribute emotions to others develops more rapidly than the ability to attribute thoughts and that the growing efficiency of hot ToM with age is specifically rooted in the growing efficiency of hot IC abilities.

## Introduction

For nearly forty years, researchers have studied the human ability to attribute mental states such as intentions, desires, beliefs, thoughts or emotions to others [1–3]. This meta-representational ability, referred to as theory of mind (ToM), has received much attention because this ability is critical for social adaptation [4–6]. Recently, studies in adults [7] and children and adolescents [8, 9] have provided evidence that the ability to attribute affectively neutral mental states such as thoughts to others (hereafter referred as cool ToM) could be dissociated from the ability to attribute affectively charged mental states such as emotions to others (hereafter referred as hot ToM). In particular, studies have shown that hot ToM can be selectively impaired despite preserved cool ToM in adults diagnosed with schizophrenia [7] or psychopathy [10]. In addition, cool and hot ToM rely on partially distinct networks [10–13], with cool ToM relying more on the dorsolateral prefrontal cortex (DLPFC) and hot ToM on the ventromedial prefrontal cortex (VMPFC). Other studies grant a broader role to the VMPFC, suggesting that it could be involved in inferential reasoning that would include the ability to attribute cool and hot mental states as well as non-mentalistic inferences such as physical states attributes [14, 15]. In particular, studies have shown that hot ToM can be selectively impaired despite preserved cool ToM in adults diagnosed with schizophrenia [7] or psychopathy [10]. In addition, cool and hot ToM rely on partially distinct networks [10–13], with cool ToM relying more on the dorsolateral prefrontal cortex (DLPFC) and hot ToM on the ventromedial prefrontal cortex (VMPFC). Finally, children and adolescents with developmental disorders display subtle differences in cool and hot ToM deficits [8, 16]. Models of ToM development have either suggested that cool ToM is a pre-requisite for hot ToM [10], or that hot ToM is a pre-requisite for cool ToM [17]. In this context, the present study aims at better describing the development of the cool and hot ToM abilities to provide evidence for one of the two theoretical models existing in the literature.

Seminal studies on cool ToM evidenced that children at age 4 can acknowledge that X has a false belief (1^st^-order ToM) and children at age 6 can acknowledge that X has a false belief regarding what Y believes [18–20]. Since then, some ingenious experimental paradigms have evidenced that this ability emerge implicitly much earlier in the first years of life [21–26]. According to Shamay-Tsoory’s model [10], hot ToM is more complex than cool ToM as it requires the additional integration of an emotional component. Therefore, the development of cool ToM would precede the development of hot ToM. In line with that view, children tend to first master the ability to attribute a false belief (cool ToM) before being able to anticipate one’s emotion (hot ToM) given one’s false belief [27]. In addition, complex emotional state inference only appears in late childhood with children successfully recognizing a faux pas around 9–11 years of age [28]. The latter development of ToM from adolescence to adulthood also conveys this asynchronous pattern where cognitive ToM is mastered sooner than its affective facet [29].

The opposite view considers that children actually build their metarepresentational abilities first through the inference of affective mental states and then with the inference of more abstract mental states [17]. For instance, children first explain other’s behaviors by referring to emotions and desires before referring to the cognitive mental states [30]. Along the same lines, preschoolers understand diverging desires (hot ToM) between people before they understand diverging beliefs between people and infants as young as 2 years old appear to be able to correctly impute and respond to others’ desire, even if their own desire is in opposition with other’s desire [17, 31, 32]. Children aged 3 to 5 perform better when asked to do affective than cognitive perspective-taking [33]. Moreover, longitudinal studies indicate that early hot ToM abilities predict latter cool ToM abilities [34, 35].

Discrepancies regarding the development of hot and cool ToM might be in part related to the fact that most of the previous studies used different tasks to assess hot and cool ToM. Thus, in the present study, we study the development of cool and hot ToM abilities using simple ToM tasks that only differ by their affective nature.

In addition, we also investigated in the present study the executive processes at play in the development of cool and hot ToM abilities because a number of studies evidence that ToM development is at least in part related to executive functions (EF) and to inhibitory control (IC) development in particular [36–39]. The development of inhibitory control has also been investigated by contrasting it when it is exerted in an affectively neutral (cool) or affectively charged (hot) contexts. These studies have drawn inconsistent conclusions suggesting either a total overlap between the two constructs [40, 41] or not [42]. One way to tackle this question is to study the relation between cool and hot IC and cool and hot ToM throughout development. For instance, some studies provided evidence that cool but not hot EF are correlated to cool ToM in toddlers from 3 to 4 years of age [43] while in children aged 5 to 12 cool executive functioning explained performance in a cool ToM task and hot executive functioning in a hot ToM task [44]. Yet, these findings are insufficient to determine precisely the role of hot and cool IC in hot and cool ToM development.

The present study thus aimed at i) characterizing the developmental pattern of cool and hot ToM in children aged 3.5 to 6.5 using two versions of the ToM task and ii) determining the relation between cool and hot ToM and cool and hot IC from 3.5 to 6.5 years of age. Age range was defined in regards to 1st and 2^nd^ order ToM development [18–20]. Children performed an adaptation of the Yoni task [13], which is a simplified test of 1^st^- and 2^nd^-order ToM, in three conditions: cool, hot and control conditions. In addition, children performed the day-night and the happy-sad tasks to assess their cool and hot IC abilities [45] and the vocabulary subtest of the Wechsler Preschool and Primary Scale of Intelligence WPPSI-IV [46] to control for individual differences in verbal abilities.

Two opposite hypotheses can be made regarding the developmental pattern of cool and hot ToM. If cool ToM is the foundation of Hot ToM, then performance in the cool condition of the Yoni task should develop faster between 3.5 to 6.5 years of age than in the hot one. If hot ToM is the foundation of cool ToM, then performance in the hot condition of the Yoni task should develop faster between 3.5 to 6.5 years of age than in the cool. In addition, we reasoned that if cool and hot IC play an exclusive role respectively in cool and hot ToM, then performance in the day-night task should be linked with the performance on the cool but not the hot condition of the Yoni task and vice versa for the performance in the happy-sad task. Finally, if cool and hot IC support cool and hot ToM development, respectively, then the effect of age on the performance in the cool and hot conditions of the Yoni task should be mediated by the performance in the day-night and the happy-sad tasks, respectively.

## Materials and method

### Participants

One hundred thirty-seven children were recruited from the same school, serving a diverse population with a wide range of economic statuses. Data regarding the socioeconomic background of the participants were not collected. For each child, written informed consent was obtained from their parent. Twenty-five children were excluded from the analysis. Two for not having succeeded in correctly identifying the pictures in the first part of the vocabulary task, 6 for not having completed all the tasks. In the Yoni task, 16 were discarded for performing at chance-level in one of the control conditions. Given that these control condition requires a non-mentalistic inference regarding a physical attribute, performing at chance, is likely to reflect a lack of motivation for the task. Note that, running the same analysis on the sample including these ‘at-chance-level’ participants (N = 128) yielded to the same findings. The final sample was thus composed of 112 children (60 girls, 52 boys; *M* = 4.97 years old, *SD* = 1.12) who were in the school year corresponding to their date of birth. Four homogeneous groups of ages were created a posteriori. The youngest group included 26 children (14 girls; M = 3.5 years old, SD = .28), the second group included 28 children (17 girls; M = 4.47 years old, SD = .35), the third group included 30 children (15 girls; M = 5.38 years old, SD = .33) and the fourth group included 28 children (14 girls; M = 6.47 years old, SD = .25). The proportion of males to females did not differ between the four groups, χ^2^(3) = .88, *p* = .83. Sample size was determined pre hoc using G*Power 3.1.9.2 [47]. The power analysis revealed that a minimum of 40 participants would be necessary within a mixed-design analysis of variance (ANOVA) with power (1- β) set at .80 and α set at .05 to detect a medium effect size of 0.25.

The internal ethical board of the Faculty of Psychology ruled that in light of the potential risks for the participants of the present study, no formal ethical approval by one of the national ethical committee was needed in agreement with the Ethical law governing human research in France. All participants were tested in accordance with national and international norms governing the use of human research participants.

### Procedure and tasks

Each child performed 4 tasks in a quiet room near their classroom. The four tasks were administered in the same order: the vocabulary subtest of the WPPSI-IV [46], the Yoni task [7], followed by the day-night and happy-sad tasks [45]. Between each task, children were invited to take a small break. For each task, the stimuli were presented on a laptop, and the experimenter coded the verbal response of each child. The experiment lasted approximately 30 minutes.

### The vocabulary task

To control for individual differences in verbal abilities that could potentially drive the developmental effect reported in the ToM task, the children performed the vocabulary subtest of the WPPSI-IV [46]. Children were first presented with three drawings and asked to name them one by one (e.g., "what is this…? " when shown a drawing of a car). One point was attributed to each drawing correctly named. Then, children were asked to give the definition of ten words (e.g., "what is a dog?"). Two points were attributed to correct answers, 1 for incomplete ones. The maximum score was 23.

### The Yoni task

Children performed 48 trials from the Yoni task adapted from Shamay-Tsoory et al. [13]. In each trial, a main character named Yoni was depicted on the center of the screen, and children had to choose out of 4 pictures displayed on the corners of the screen that corresponded to the statement. The statement involved a cool mental state (e.g., “Yoni thinks…’”), a hot mental state (e.g., “Yoni loves…”), or a nonmentalistic control statement involving a physical attribute (e.g., “Yoni is close to…”). The mental state was to be inferred from Yoni’s eye gaze and his mouth expression. The children provided their answer either by pointing to the pictures or naming the object or the character depicted in the picture. The first half of the trials consisted of 1^st^-order ToM statements, e.g., “Yoni is thinking of….”, and the second half consisted of 2^nd^-order ToM statements, such as “Yoni is thinking of the object that …. is also thinking about”. The children performed 16 trials per condition (i.e., 16 cool ToM, 16 hot ToM, 16 control). Each child performed one block of 24 first-order ToM trials before a block of 24 second-order ToM trials. The trials in each block were presented in a pseudorandomized order. Note that previous studies evidenced (a) that 1^st^-order trials were performed more efficiently than in 2^nd^-order trials [13, 16] and (b) that cool and hot ToM trials rely on different networks classically associated with cognitive or affective ToM [7, 10, 11, 13]. Taken together, findings from previous studies provide evidence that the Yoni task can assess 1^st^ and 2^nd^ order ToM in affectively (hot) and non-affectively charged (cool) contexts.

### The day-night and the happy-sad tasks

Children performed adaptations of the day-night and the happy-sad tasks [45]. In the day-night task, a picture of the moon or the sun was displayed on the computer screen, and the child was instructed to say “day” when shown the moon and “night” when shown the sun. A total of 24 trials (i.e., 12 sun and 12 moon) were presented in the same pseudorandomized order for each child. In the happy-sad task, a picture of a happy or a sad face was displayed on the computer screen, and the child was instructed to say “happy” when shown the sad face and “sad” when shown the happy face. A total of 24 trials (i.e., 12 happy and 12 sad faces) were presented in the same pseudorandomized order for each child. The experimenter pressed the left or the right button of the mouse pad to indicate the response provided by the children: right button for sad and night responses or left for happy and day responses.

## Results

To compute ARs in the Yoni task that included 48 trials in total, we divided the number of correct responses obtained by each child for each condition (i.e., hereafter referred to as Cool 1^st^, Cool 2^nd^, Hot 1^st^, Hot 2^nd^, Control 1^st^, Control 2^nd^) by the total number of trials of that condition (i.e., 8 trials for each) leading to an accuracy rate between 0 and 1. Following the same principle, we divided the number of correct responses obtained by each child in the day-night and the happy-sad tasks by the total number of trials in each task (i.e., 24 trials per task: cool IC, Hot IC) leading to an accuracy rate between 0 and 1 for each task (i.e., hereafter referred as cool IC and hot IC). The vocabulary score was scaled by diving each child’s total score by the maximum score (i.e., 23) leading to an accuracy rate between 0 and 1. Effect size are reported for every analyzes using partial eta square and cohen’s d. To go further, we also report Bayesian statistics. The Bayes factor (BF) allows to compare two hypothesis and to assess how many times more likely the data is under one hypothesis relatively to the other [48]. Here, we report two types of BFs. The first one assesses how many times the data is more likely under a model including a factor compared to the null model (BF_10_). The second one assesses how many times the data is more likely to occur under a model including the factor of interest relatively to equivalent models stripped of the effect (BF_incl_). The JASP software (version 0.14.1) was used to conduct all analyses.

### The Yoni task

To determine the developmental pattern of cool and hot ToM, we ran a repeated measures Anova on the ARs in the Yoni task with Age as a between-participants factor (Age: 3.5 vs. 4.5 vs. 5.4 vs. 6.5 years of age), with Order (Order: 1^st^ vs 2^nd^) and Condition (Condition: cool vs. hot vs. control) as within-participants factors, and with the AR in the vocabulary task as a covariate (see Table 1).We found a main effect of Age, *F*(3, 108) = 38.52, *p* < .001, η_p_^2^ = .52, BF_incl_ = 2.91e^6^, with older children performing better than younger children. Bonferroni corrected post hoc comparisons revealed that performance in the Yoni task differed only between 3.5-year-old and 4.5-year-old children, *t*(108) = 5.98, *p* < .001, *d* = .57, BF_10_ = 2.12e7, and between 4.5-year-old and 5.4-year-old children, *t*(108) = 2.76, *p* = .02, *d* = .26, BF_10_ = 25.52. We also found a main effect of Order with children performing better in the 1^st^-order than in 2^nd^-order trials, *F*(1, 108) = 146.15, *p* < .001, η_p_^2^ = .58, BF_incl_ = 9.67e^34^. The main effect of Condition was also significant, *F*(2, 216) = 138.65, *p* < .001, η_p_^2^ = .56, BF_incl_ = 2.91e^42^, with children performing better in the control than in the hot conditions, *t*(216) = 9.36, *p* < .001, *d* = .88, BF_10_ = 8.88e^14^, and better in the hot than the cool condition, *t*(216) = 7.25, *p* < .001, *d* = .67, BF_10_ = 5.65e^11^. Finally, we found a significant two-way interaction between Age and Condition, *F*(6, 216) = 8.71, *p* < .001, η_p_^2^ = .20, BF_incl_ = 4.94e^6^, and between Condition and Order, *F*(2, 216) = 12.23, *p* < .001, η_p_^2^ = .10, BF_incl_ = 148.48, and a three-way interaction between Age, Condition and Order, *F*(6, 216) = 6.06, *p* < .001, η_p_^2^ = .15, BF_incl_ = 106.35. Note that running a mixed-model with ARs as the dependent variable, Condition and Order as fixed effects (dummy coded) and participants as a random effect yielded to the same triple interaction between Age, Order and Condition *F*(6, 540) = 4.37, *p* < .001. Additional analysis, adding Gender as a between-participants factor is provided in the supplementary materials.

**Table 1:** Mean ARs (%) in 1^st^-order trials and 2^nd^-order trials of the cool, hot, and control conditions of the Yoni task for each age group. Standard deviations appear in parentheses.

	1^st^-order trials			2^nd^-order trials		
Age group	Cool	Hot	Control	Cool	Hot	Control
3.5 years	45.2 (25.3)	56.3(24.8)	92.3 (10.7)	26 (16.2)	42.3 (22.1)	67.3 (22.1)
4.5 years	69.6 (29.2)	83 (21.6)	98.2 (4.45)	50 (21.2)	67.9 (19.4)	79 (23.1)
5.4 years	90 (21.1)	92.1(19.6)	95 (12.5)	59.6 (24.9)	72.9 (19.4)	90.8 (16.1)
6.5 years	95.5(14.5)	97.3(14.2)	97.3 (6.23)	61.6 (25)	79.9 (17.5)	93.3 (11.5)

To specify the three-way interaction, we conducted repeated measures ANOVAs with Age as a between-participants factor (Age: 3.5 vs. 4.5 vs. 5.4 vs. 6.5 years of age) and Condition (Condition: cool vs. hot vs. control) as a within-participants factor separately on the 1^st^- and 2^nd^-order trial ARs with the AR in the vocabulary task as a covariate. ANOVA on 1^st^-order trial ARs revealed a significant interaction between Condition and Age, *F*(6, 216) = 17.69, *p* < .001, η_p_^2^ = .33, BF_incl_ = 4.23e^13^, a significant main effect of the Condition *F*(2, 216) = 62.95, *p* < .001, η_p_^2^ = .37, BF_incl_ = 9.77e^12^, and a significant main effect of Age, *F*(3,108) = 25.64, *p* < .001, η_p_^2^ = .42, BF_incl_ = 5.62e^4^.

**Fig 1:**
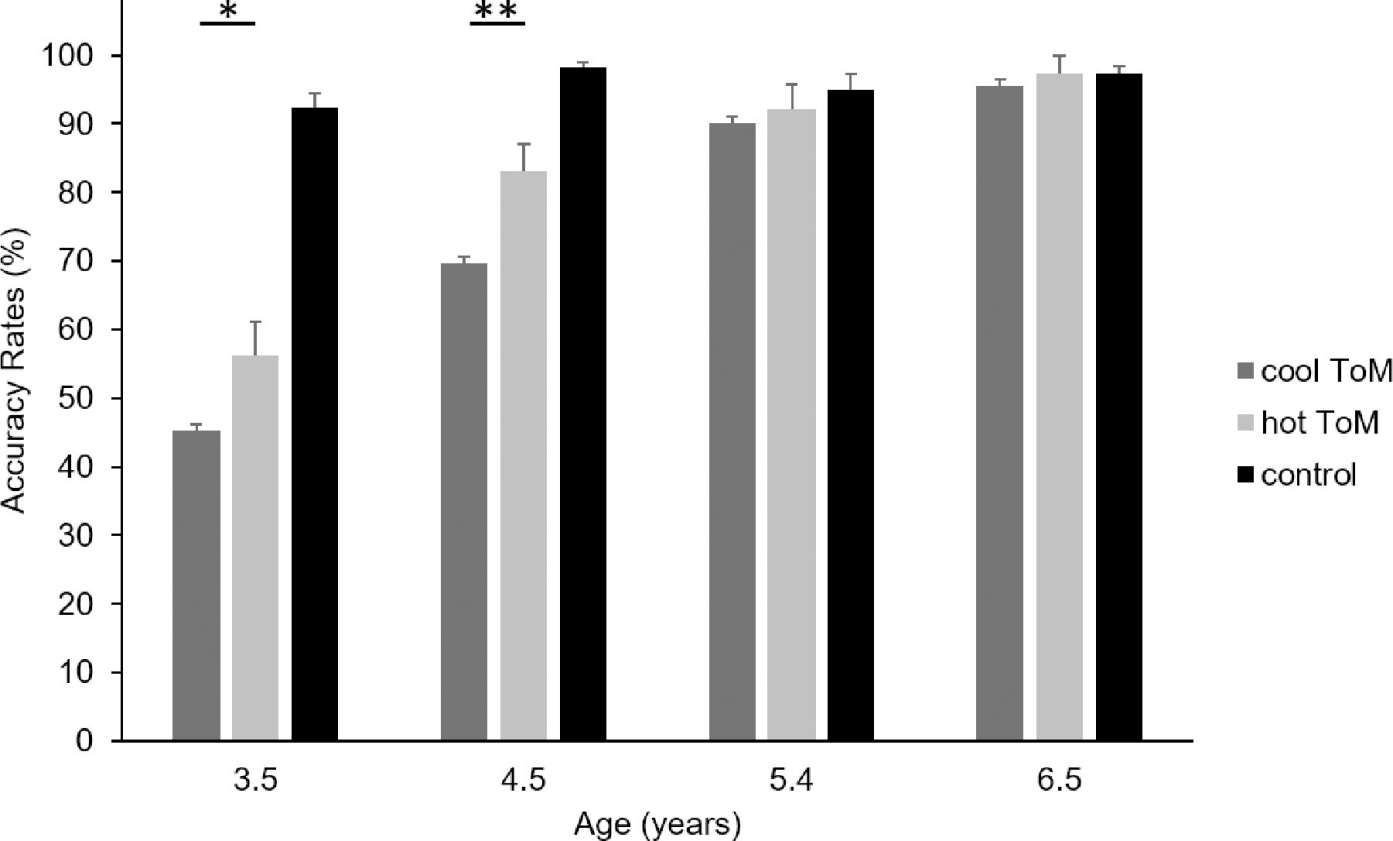
Mean ARs (%) for each age group in the 1st-order trials of the cool, hot and control conditions of the Yoni task. Error bars denote standard error of the mean. *p < .05. ** *p* < .005.

We characterized the interaction on the 1^st^-order trial ARs using Bonferroni correction for multiple comparisons (see Fig 1). We found that 3.5- and 4.5-year-old children were more accurate in performing hot than cool 1^st^-order trials, *t*(216) = 2.86, *p* = .04, for 3.5-year-old children and *t*(216) = 3.59, *p* = .003, for 4.5-year-old children; in contrast, children aged 5.4 and 6.5 years were as efficient in performing 1^st^-order trials in the hot than in the cool conditions of the Yoni task, *t*<1, for 4.5-year-old children and *t*<1, for the 6.5-year-old children (see Table 1).

**Fig 2:**
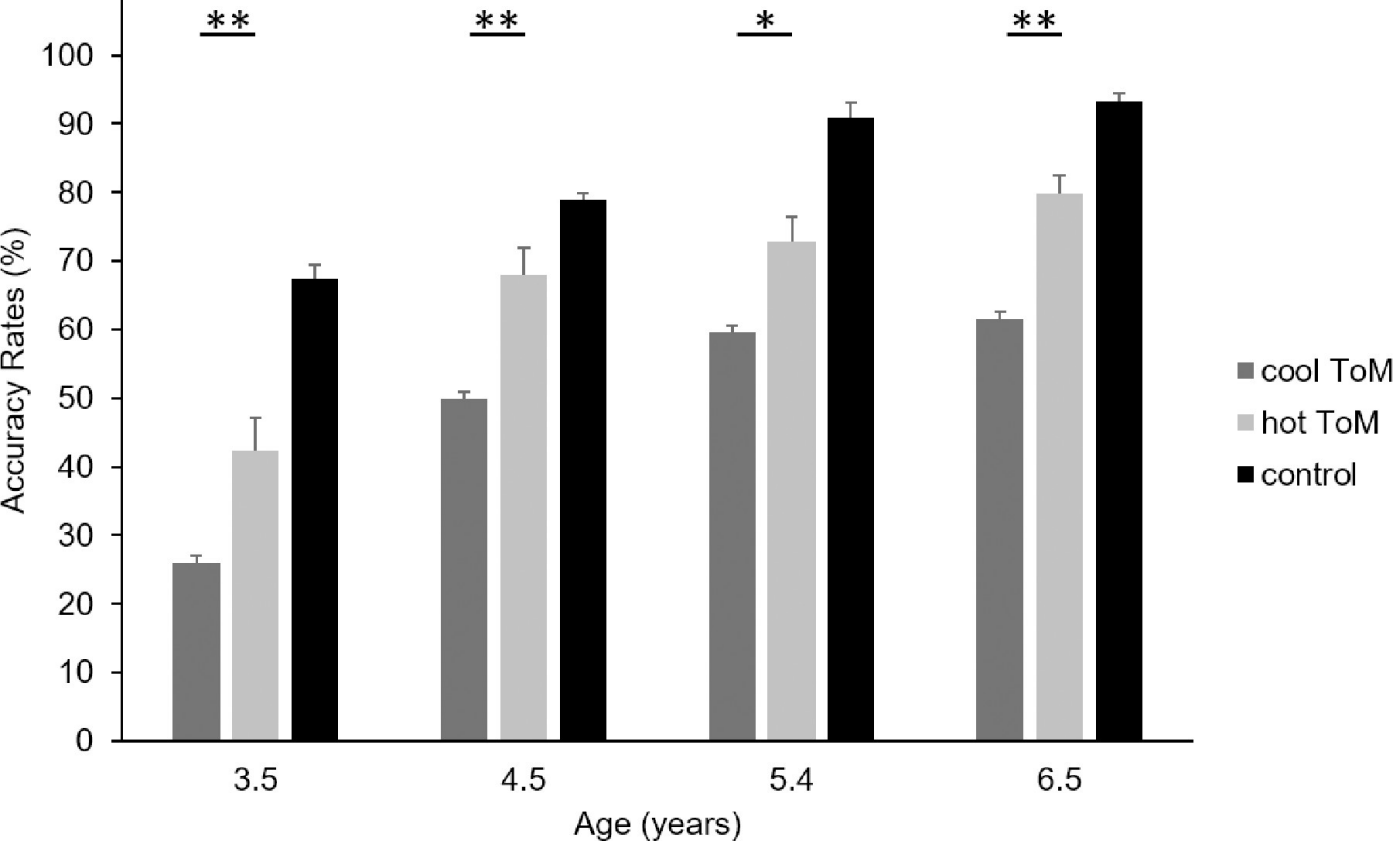
Mean ARs (%) for each age group in the 2nd-order trials of the cool, hot and control conditions of the Yoni task. Error bars denote standard error of the mean. *p < .05. ** *p* < .005.

ANOVA on 2^nd^-order trial ARs revealed a main effect of Condition, *F*(2, 216) = 103.99, *p* < .001, η_p_^2^ = .49, BF_incl_ = 2.04e^30^, with children of all ages performing better on hot than on cool 2^nd^-order trials: *t*(216) = 3.41, *p* = .006, for 3.5-year-old children, *t*(216) = 3.87, *p* = .001, for 4.5-year-old children, *t*(216) = 2.99, *p* = .024, for children aged 5.4 years, and *t*(216) = 3.97, *p* < .001, for children aged 6.5 years (see Fig 2). The main effect of Age was also significant, *F*(3, 108) = 27.80, *p* < .001, η_p_^2^ = .44, BF_incl_ = 7.37e^3^, but there was no interaction between Condition and Age, *F*(6, 216) = 1.09, *p* = .371, BF_incl_ = 0.07.

### The day-night and happy-sad tasks

To determine the developmental pattern of cool and hot IC, we ran a repeated measures Anova with Age as a between-participants factor (Age: 3.5 vs. 4.5 vs. 5.4 vs. 6.5 years of age), and with Type of IC (Type of IC: cool vs. hot) as a within-participants factor on the ARs. This analysis revealed a main effect of Age, *F*(3, 108) = 24.13, *p* < .001, η_p_^2^ = .40, BF_incl_ = 1.84e^9^, with better performance in 4.5- than in 3.5-year-old children, *t*(108) = 5.11, *p* < .001, *d* = .48, BF_10_ = 8.18e^3^, (Bonferroni-corrected post hoc comparisons). We found a main effect of the Type of IC, *F*(1, 108) = 27.25, *p* < .001, η_p_^2^ = .20, BF_incl_ = 1.20e^4^, with children committing fewer errors in the cool than in the hot IC task, *t*(108) = 5.22, *p* < .001, *d* = .49, BF_10_ = 1.23e^4^. Finally, there was no interaction between the Age and the Type of IC, *F*(3, 108) = 1.11, *p* = .35, BF_incl_ = 0.87 (see Table 2). Note that running a mixed-model with ARs as the dependent variable, Age and Type of IC as fixed effects (dummy coded) and participants as a random effect yielded to the same effects: a main effect of Age *F*(3, 108) = 24.13, *p* < .001, a main effect of Type of IC *F*(1, 108) = 27.25, *p* < .001 but no significant interaction between Age and the Type of IC *F*(3, 108) = 1.11, *p* = .35. Additional analysis, adding Gender as a between-participants factor is provided in the supplementary materials.

**Table 2:** Mean ARs (%) in the day-night and happy-sad task for each age group. Standard deviations appear in parentheses.

Age group	Day-Night task	Happy-Sad task
3.5 years	57.8 (27.7)	42 (28.8)
4.5 years	76.6 (19.2)	70.5 (18.2)
5.4 years	84.2 (12)	74.6 (18.3)
6.5 years	91.8 (10.7)	83.2 (18.3)

### Multiple linear regression and causal mediation analyses

To study how age, vocabulary, hot IC and cool IC predicted the ARs in the four levels of interest, we performed a mixed model on the ARs of Cool 1^st^ order, Cool 2^nd^ order, Hot 1^st^ order, Hot 2^nd^ order combined as a dependent variable. For the purpose of the following analyses we computed the age z-scores for each participant by subtracting the mean age of the population to its age and dividing this difference by the standard deviation of age over the population. As fixed effects we used the age z-scores, ARs in the vocabulary task, IC cool ARs, IC hot ARs and their respective interaction with a new dummy coded variable specifying the four levels (Cool 1^st^ order, Cool 2^nd^ order, Hot 1^st^ order, Hot 2^nd^ order). Participants were used as a random effect to account for the mixed design. To obtain main effects, Type II analysis-of-variance using Wald χ^2^ tests was performed on the model. We found a main effect of age on ARs (independently of the level): *χ*^*2*^ (1, 112) = 15.05, *p* < .001, an interaction between the level and age: *χ*^*2*^ (5, 112) = 26.57, *p* < .001) and a trending interaction between the level and hot IC χ^2^ (110, 112) = 129.78, *p* < .10. To further investigate the interactions and understand how much of the variance is explained in each level, we performed independent multiple linear regression on each level. The four predictors explained 39.6% of the variance in Cool 1^st^ order trials, *F*(4, 107) = 17.50, *p* < .001, BF_10_ = 2.44e^8^, 27.7% of the variance in Cool 2^nd^ order trials, *F*(4, 107) = 10.3, *p* < .001, BF_10_ = 3.41e^4^, 34.2% of the variance in Hot 1^st^ order trials, *F*(4, 107) = 13.9, *p* < .001, BF_10_ = 3.43e^6^, and 39.2% of the variance in Hot 2^nd^ order trials, *F*(4, 107) = 17.3, *p* < .001, BF_10_ = 1.83e^8^. The participants’ age z-scores was a predictor of ToM performance: *β* = .48, *t*(111) = 4.27, *p* < .001, BF_incl_ = 1.20e^4^ in cool 1^st^-order trials; *β* = .44, *t*(111) = 3.58, *p* < .001, BF_incl_ = 368.63 in cool 2^nd^-order trials; *β* = .41, *t*(111) = 3.49, *p* < .001, BF_incl_ = 497.57 in Hot 1^st^ order trials and *β* = .40, *t*(111) = 3.55, *p* < .001, BF_incl_ = 190.73 in Hot 2^nd^ order trials. Performance in the hot IC task was also a predictor of Hot 2^nd^ order trials, *β* = .31, *t*(111) = 2.93, *p* = .004, BF_incl_ = 9.12.

Because both age and hot IC predicted performance in the 2^nd^-order trials of the hot ToM condition, we performed a causal mediation analysis to determine whether hot IC mediated the relation between age and hot 2^nd^-order ToM ability. Using a classic procedure in causal mediation analysis [49], we tested (a) the total effect of age z-scores (predictor variable) on the performance in the in the 2^nd^-order trial of the hot ToM condition of the Yoni task (outcome variable) (path c), (b) the relation between age z-scores (predictor variable) and the performance in the hot IC task (mediator variable) (path a), (c) the relation between the performance on the hot IC task (mediator variable) and the performance in the 2^nd^-order trial of the hot ToM condition of the Yoni task (outcome variable) while controlling for the age z-scores (predictor variable) (path b), and (d) the relation between the predictor variable and the outcome variable while controlling for the mediator (path c’). We used a nonparametric bootstrapping method to test the significance of the indirect effect (path c’) [50–52]. The generated distribution enabled us to compute the 95% confidence interval (CI) of the indirect effect. A 95% CI of the indirect effect that did not include 0 demonstrated that the indirect effect was significant.

**Fig 3:**
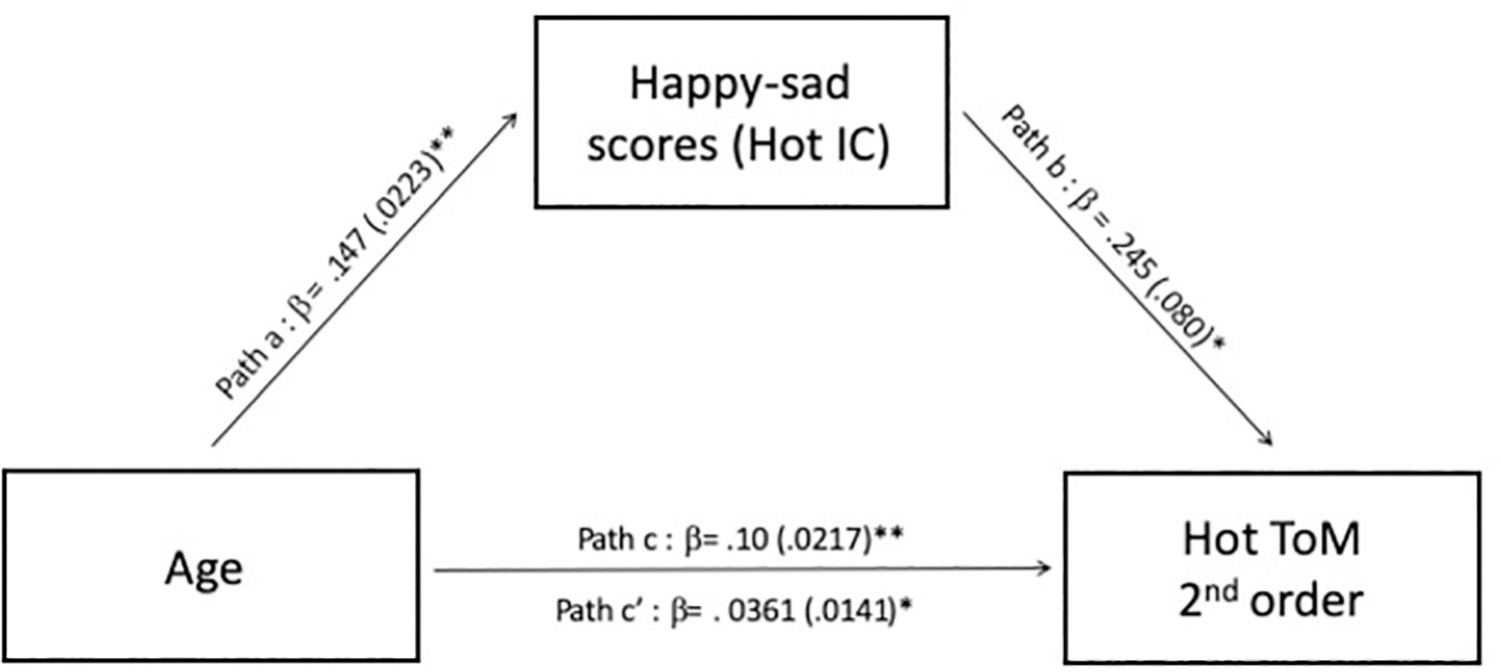
Mediation analysis for the hot 2nd-order ToM condition of the Yoni task. Paths a, b, and c report the unstandardized beta weight for the direct effect of age z-scores on happy-sad scores (hot IC), the direct effect of the happy-sad scores on 2nd-order trials of the hot ToM ARs and the direct effect of age z-scores on 2nd-order trials of the hot ToM ARs, respectively. Path c’ reports the unstandardized beta weight of the indirect effect of age on 2nd-order trials of the hot ToM ARs when the happy-sad scores (hot IC) is added to the model. *** Significant at the .001 level and ** significant at the .01 level.

As shown in Fig 3, the mediation analysis revealed the following: (a) age z-scored was associated with performance on the hot 2^nd^-order ToM condition of the Yoni task (path c), *β =* .136 (.019), *t* = 7.31, *p* < .001; (b) age z-scored was associated with performance on the hot IC task (path a), *β =* .147 (.020), *t* = 7.29, p < .001; (c) hot IC performance was associated with performance on the hot 2^nd^-order ToM condition of the Yoni task while controlling for age (path b), *β =* .245 (.09), *t* = 2.88, *p* = .005 (path b); and (d) age z-scored was associated with performance on the hot 2^nd^-order ToM while controlling for performance on the hot IC task *β* = .100 (.022), t = 4.56, p < .001 (path c’). The effect of age z-scores on the performance on the hot 2^nd^-order ToM condition of the Yoni task was partially mediated by hot IC performance. The indirect effect was *β =* (.147)*(.245) = .036 (.013), 95% CI [.013, .08], *p* = .007 and represented 26.5% of the total effect of age z-scores on the performance on the hot 2^nd^-order ToM condition of the Yoni task.

## Discussion

The goal of the present study was to determine (a) the developmental pattern of cool and hot ToM abilities from 3.5 to 6.5 years of age using two conditions of the same task and (b) the role of cool and hot IC in the development of cool and hot ToM abilities. As expected and in line with previous studies, we found that 1^st^- and 2^nd^-order ToM abilities improved from 3.5 to 6.5 years of age [see 19, 53] and that 1^st^-order ToM abilities developed sooner than 2^nd^-order ToM abilities [18–20]. Importantly, we found that children were more efficient at attributing affectively charged mental states (i.e., emotions) than affectively neutral mental states (i.e., thoughts). This was true for 3.5- and 4.5-year-old children in the 1^st^-order ToM and through 6.5 years of age for the 2^nd^-order ToM. Consistently with several studies carried out on typically developing children [17, 31–33] and atypically developing children [8], our results demonstrate that hot metarepresentational abilities develop faster than cool ones. These results also fit the neurodevelopmental model of empathy showing that the affective component of empathy would develop before its cognitive component [54–56]. Indeed, the ability to feel as somebody else and the ability to show empathic concern would build up first on bottom-up processes allowing emotion understanding and shared emotional experience and then on more complex top-down evaluation including inter-individual appraisal of intentions. This was for instance evidenced by a shift in the neural response to the viewing of intented painful situations from childhood to adulthood from the medial part to the lateral part of the VMPFC [56].

By evidencing that cool and hot ToM have a specific development with an earlier development of hot ToM than of cool ToM, the present findings disentangle the two main competing models. Indeed, our data support the view that hot ToM might be a pre-requisite for cool ToM [17] and not the other way around [10]. This could be due to the fact that emotional mental states are actually easier to grasp than cognitive ones as they are more often associated with clear external cues [17]. For instance, children might first infer that if somebody smiles it is likely because of a positive affective mental state. Then, they could gradually grant to others cognitive mental states such as thoughts that do not translate into facial expressions. Put differently, the development of these two facets of ToM would be interrelated such that hot ToM abilities might predict later cool ToM abilities [34, 35]. However, the developmental dynamic of cool and hot ToM might be different in atypically developing children and other critical abilities such as IC could favor the development of cool ToM [57]. For instance, children with callous-unemotional traits develop efficient cool ToM abilities thanks to their IC abilities, despite having difficulties in hot ToM.

By assessing the association between cool and hot ToM and cool and hot IC for the first time in children of different ages, we provided evidence that the growing hot ToM abilities with age was specifically related to hot IC abilities but not cool ToM abilities. Thus, our results provide further support for the role of EF in ToM development [e.g., 28–31], suggesting that different forms of IC might support different forms of ToM. In addition, we found that hot IC efficiency mediated the link between age and hot ToM, which suggested that the development of hot ToM abilities was partially rooted in the development of hot IC efficiency. Finally, demonstrating a link between ToM and IC using a ToM task with low executive demand upholds the idea that IC could participate in the emergence of ToM and not only be a component of its expression [37, 57]. However, in contrast to what we hypothesized, we found no relation between performance in 1^st-^ and 2^nd^-order cool ToM trials of the Yoni task and performance in the day-night task. Thus, in contrast to previous studies [58, 59], cool IC and cool ToM abilities were not related in our study. We suspect that the lack of correlation between cool IC and cool ToM abilities could be due either to the specificity of the cool IC task and/or of the cool version of the Yoni task, which is a cool ToM task with low executive demands unrelated to ToM processes, unlike other cool ToM tasks [60, 61].

It is of note that the order of the tasks in the present study was maintained constant across participants. Every child started by the vocabulary task, followed by the Yoni task (1^st^ order trials before 2^nd^ order ones), to end up with the day-night and the happy-sad tasks. This choice was made to minimize interindividual variability caused by an order effect to better investigate the links between inhibition and theory of mind. In addition, the fact that the IC tasks were performed after the ToM tasks prevent from any transfer effect from the IC tasks to the ToM tasks. Yet, such unrandomized design could affect the results and further studies should allow to control for potential task order effects [62]. In addition, one could regret the absence of data regarding the socio-economical background of the participants included in the present experiment. In conclusion, we provide the first evidence that i) typical development is characterized by an earlier development of the ability to attribute affectively charged than affectively neutral mental states to others and ii) that the growing hot ToM abilities with age are partly rooted in the growing hot IC efficiency. Given that (a) emotion understanding is pivotal for children’s social adjustment and academic achievement [62–64], (b) ToM is a key factor in promoting learning [65–67], and (c) several developmental disorders evidenced subtle differences in cool and hot ToM impairment [8, 16], future studies should test the impact of specific cool and hot IC training on the improvement in cool and hot ToM performance as well as on school outcomes [68, 69].

## Supporting information

S1 Data Supplementary analyses. (DOCX)
