# Supplementary material for: Development of cool and hot theory of mind and cool and hot inhibitory control abilities from 3.5 to 6.5 years of age
Source: PLoS One. 2022 Jan 27;17(1):e0262251. doi: 10.1371/journal.pone.0262251 (PMC8794116; doi:10.1371/journal.pone.0262251)
Supplement: S1 Data — (DOCX) [file pone.0262251.s001.docx]

**S1. Supplementary analyses.**

We ran a repeated measures Anova with Age and Gender as between-participant factors, and with Condition and Order as within-participant factors, while controlling for the ARs in the vocabulary task. This analysis evidenced an effect of Gender *F*(1, 103) = 5.82, *p* = .018, *η_p_²* = .054 showing that males outperformed females, and an interaction between Age-group, Gender, Condition and Order *F*(6, 206) = 2.25, *p* = .04, *η_p_²* = .061. Given that the main result of the current study lies in the triple interaction between Age-group, Condition and Order we did investigate it separately for males and females.

The main findings of the current study are actually consistent across gender showing a significant triple interaction, respectively for female participants, *F*(6, 112) = 3.52, *p* = .003, *η_p_²* = .159, and for male participants *F*(6, 96) = 5.04, *p* < .001, *η_p_²* = .239

More precisely, we found for female participants: a main effect of Age *F*(3, 56) = 18.34, *p* < .001, *η_p_²* = .496, a main effect of Order *F*(1, 56) = 58.57, *p* < .001, *η_p_²* = .511, a main effect of Condition *F*(2, 112) = 84.63, *p* < .001, *η_p_²* = .602, an interaction between Condition and Age *F*(6, 112) = 6.12, *p* < .001, *η_p_²* = .247, and an interaction between Condition and Order F(2,112) = 5.12, p < 0.01, *η_p_²* = .084.

For male participants, we evidenced a main effect of Age *F*(3, 48) = 26.32, *p* < .001, *η_p_²* = .622, a main effect of Order *F*(1, 48) = 97.12, *p* < .001, *η_p_²* = .669, a main effect of Condition *F*(2, 96) = 51.95, *p* < .001, *η_p_²* = .602, and an interaction between Condition and Age *F*(6, 96) = 2,68, *p* = .019, *η_p_²* = .143, and an interaction between Condition and Order *F*(2, 96) = 8.39, *p* < .001, *η_p_²* = .149.

Table A: Mean ARs (%) in 1^st^-order trials and 2^nd^-order trials of the cool, hot, and control conditions of the Yoni task for each age group and depending on the gender of participants. Standard deviations appear in parentheses.

|  |  | 1^st^-order trials | | | 2^nd^-order trials | | |
| --- | --- | --- | --- | --- | --- | --- | --- |
| Age group | Gender | Cool | Hot | Control | Cool | Hot | Control |
| **3.5 years** | *Female* | 42.9 (21.2) | 47.3 (20.3) | 92 (11.6) | 18.8 (9.5) | 42.9 (25.3) | 68.8 (24.4) |
|  | *Male* | 47.9 (30.1) | 66.7 (26.3) | 92.7 (9.9) | 34.4 (18.6) | 41.7 (18.7) | 65.6 (20) |
| **4.5 years** | *Female* | 64.7 (30.1) | 80.9 (22.1) | 97.8 (4.9) | 44.1 (18.8) | 59.6 (19) | 72.8 (24.3) |
|  | *Male* | 77.3 (27.3) | 86.4 (21.3) | 98.9 (3.8) | 59.1 (22.4) | 80.7 (11.7) | 88.6 (18.1) |
| **5.4 years** | *Female* | 85 (28.4) | 89.2 (26.2) | 94.2 (16.3) | 63.3 (27.3) | 69.2 (23.6) | 91.7 (13.9) |
|  | *Male* | 95 (7.9) | 95 (9.2) | 95.8 (7.7) | 55.8 (22.6) | 76.7 (14.1) | 90 (18.4) |
| **6.5 years** | *Female* | 93.8 (20.1) | 94.6 (20) | 97.3 (5.3) | 61.6 (25.7) | 81.2 (20.1) | 92.9 (14.5) |
|  | *Male* | 97.3 (5.3) | 100 (0) | 97.3 (7.2) | 61.6 (25.2) | 78.6 (15.1) | 93.8 (8.1) |

We ran a repeated measure Anova with Age and Gender as between-participant factors, and with Type of IC as a within-participant factor. Gender was not significant and did not interact either with Age or Type of ICs, *Fs* < 1. Consistently with the results reported in the manuscript, this analysis evidenced an effect of Type of IC *F*(1, 104) = 25.31, *p* < .001, *η_p_²* = .20 and an effect of Age *F*(3, 104) = 23.47, *p* < .001, *η_p_²* = .404.

Table B: Mean ARs (%) in the Day-Night and in the Happy-Sad task for each age group and depending on the gender of participants. Standard deviations appear in parentheses.

| Age group | Gender | Day-Night task | Happy-Sad task |
| --- | --- | --- | --- |
| **3.5 years** | *Female* | 57.4 (29.8) | 39 (29.2) |
|  | *Male* | 58.3 (26.4) | 45.5 (29.2) |
| **4.5 years** | *Female* | 75.5 (19) | 67.4 (18.4) |
|  | *Male* | 78.4 (20.4) | 75.4 (17.7) |
| **5.4 years** | *Female* | 86.1 (10.6) | 79.7 (10.1) |
|  | *Male* | 82.2 (13.2) | 69.4 (23.2) |
| **6.5 years** | *Female* | 91.7 (13) | 83 (21.9) |
|  | *Male* | 92 (8.4) | 83.3 (14.8) |
